# A Neurologist's Guide to TNF Biology and to the Principles behind the Therapeutic Removal of Excess TNF in Disease

**DOI:** 10.1155/2015/358263

**Published:** 2015-07-22

**Authors:** Ian A. Clark, Bryce Vissel

**Affiliations:** ^1^Research School of Biology, Australian National University, Acton, Canberra, ACT 2601, Australia; ^2^Neurodegeneration Research Group, Garvan Institute, 384 Victoria Street, Sydney, NSW 2010, Australia

## Abstract

Tumor necrosis factor (TNF) is an ancient and widespread cytokine required in small amounts for much physiological function. Higher concentrations are central to innate immunity, but if unchecked this cytokine orchestrates much chronic and acute disease, both infectious and noninfectious. While being a major proinflammatory cytokine, it also controls homeostasis and plasticity in physiological circumstances. For the last decade or so these principles have been shown to apply to the central nervous system as well as the rest of the body. Nevertheless, whereas this approach has been a major success in treating noncerebral disease, its investigation and potential widespread adoption in chronic neurological conditions has inexplicably stalled since the first open trial almost a decade ago. While neuroscience is closely involved with this approach, clinical neurology appears to be reticent in engaging with what it offers patients. Unfortunately, the basic biology of TNF and its relevance to disease is largely outside the traditions of neurology. The purpose of this review is to facilitate lowering communication barriers between the traditional anatomically based medical specialties through recognition of shared disease mechanisms and thus advance the prospects of a large group of patients with neurodegenerative conditions for whom at present little can be done.

## 1. Introduction

Nearly forty years ago the term TNF appeared in the literature. First, to set a common source of confusion to rest, we note that the synonym TNF*α*, still often seen, was an innovation that has been obsolete for some years. In 1975 the name TNF was given to a novel function of a semipurified peptide detected in the serum of mice receiving parenteral bacterial lipopolysaccharide (LPS) several weeks after they had been infected with Bacillus Calmette-Guérin (BCG), an attenuated strain of* Mycobacterium bovis*. On transfer to mice bearing transplanted sarcomas a purified form of this peptide caused necrosis of these tumors equally well as did LPS, but it was devoid of detectable LPS [1, 2]. Though named for this tumor-killing activity, in the event the peptide TNF proved to be as pleiotropic as any cytokine, being very widely distributed across biology, important in physiology, and in excess incriminated in innate immunity and disease pathogenesis. More recently its activities have been realized to include the chronic neuroinflammatory disease states seen in survivors of traumatic brain injury (TBI) and stroke, conditions that impose great personal, social, and monetary burden on individual families and society. In other words, both the infectious and noninfectious diseases seen in hospitals every day comprise the visible end of the spectrum of the biology of this cytokine.

TNF neutralization by biological therapy (i.e., by* in vitro* biologically generated specific anti-TNF agents) has become an enormous success story in a number of chronic inflammatory diseases outside the brain but curiously has not yet been taken seriously enough by the relevant medical specialists for its possible widespread adoption to be objectively examined for the chronic neuroinflammatory disease states. A 2010 editorial marking the 40th anniversary of the journal* Stroke* laments the dearth of advances leading to new targets for poststroke therapy, treatment ideas for which have stagnated [3]. Its theme is to urge the field to break down its silo mentality and embrace new ideas and approaches from other disciplines and diseases. Neutralizing excess cerebral TNF is a good example. Although clinical neurologists are aware of the concept, they largely retain, in the absence of first-hand experience or observation, what they apparently believe is healthy skepticism towards it. As we shall see, this could well arise from insufficient basic knowledge of cytokine biology. In contrast, many neuroscientists, with the advantage of wider basic knowledge of cytokine function, including its roles in homeostasis and plasticity, are on record as having independently observed the effects of neutralizing excess TNF in chronic neuroinflammatory diseases in patients [4–6] and noted its plausible clinical application. The aim of this review is to provide sufficiently broad knowledge of TNF biology for presently skeptical neurologists to engage with this approach and thus make informed decisions about trialing and treating these diseases in ways that parallel well-established treatments for important chronic systemic inflammatory illnesses.

## 2. TNF in Biology, including Brain Development, Physiology, and Neuroplasticity

The capacity to make the peptide TNF appeared extremely early in biological evolution and has been scrupulously retained. The essential elements of the TNF molecule that confer function evidently have varied little, in that cells from a number of* Acropora* spp., the genus to which the major reef-builders such as staghorn corals belong, carry receptors that recognize human TNF [7]. These authors also showed that a form of TNF made by* Acropora* spp. recognizes the TNF receptors on a well-known human cell line and is implicated in coral pathology. Hence TNF, essentially as we know it now, predates bilateral symmetry. Not surprisingly, therefore, this peptide is involved in physiology [8] and disease [9, 10] of more complex creatures such as fish, and in physiology and disease in as many birds and mammals as the availability of specific reagent has allowed to be tested.

As predictable from this ubiquity, TNF is one of the cytokines that have many essential roles in normal tissues, often involving homeostasis and functional plasticity. Arguments for this general principle applying to the brain [11] as well as the rest of the body grew from seminal 1994 observations that physiological levels of TNF are necessary for normal neuron function, with a loss or gain of TNF being pathological [12]. Examples abound. TNF excess has been demonstrated to be instrumental in sciatic nerve crush, as demonstrated by enhanced axon regeneration after treatment with etanercept [13]. TNF and at least two other members of the TNF superfamily of cytokines [14, 15] mediate neurite outgrowth. Regarding normal fetal development of nociception, TNF signalling controls neurite growth, survivability, excitability, and cell differentiation mediated by nerve growth factor [16]. TNF mediates postinjury, hypothermia-induced, neurite outgrowth [17], as well as the loss of cortical dendritic spine density, plus associated memory loss, in a mouse model of congestive heart failure [18]. Its biological influence spans generations, with a requirement for adequate maternal TNF to induce, in milk, the chemokines needed for normal hippocampal development and memory in offspring [19]. TNF is released during physiological neuronal activity and plays a crucial role in regulating the strength of normal synaptic transmission [20]. It is also involved in normal neurotransmission via modulating excitatory inputs [21], trafficking of AMPA receptors [22], homeostatic synaptic scaling [23, 24], and long-term potentiation [25], and it maintains normal background levels of neurogenesis [26–28]. Mitochondrial function depends on TNF [29], as does regulation of the neurotransmitter, orexin [30], which, as recently reviewed [31], controls sleep, motor control, focused effort, appetite, and water intake. TNF also regulates neuronal type-1 inositol trisphosphate receptors (IP3R), which are central to neuronal Ca^++^ homeostasis, and thus the ionic signaling cascades on which normal function of these cells depends [32]. Clearly, all these functions are vulnerable to TNF being outside its physiological range, as outlined in the next section. Moreover, such normal regulatory changes do not indicate inflammatory activity but are part of normal physiology. As will be noted, this has important practical implications.

## 3. TNF in the Biology of Both Infectious and Noninfectious Diseases

Awareness of the capacity of excess TNF to cause disease arose from parallels between our work on BCG, LPS, and malaria [33, 34] and earlier work of the discoverers of TNF [1], who worked in a tumor context. This led to a collaboration that generated the then novel, but now widespread, argument that enhanced TNF generation is central to innate immunity, whereas its excessive production triggers major infectious disease [35, 36]. Definitive evidence, made possible by the advent of cloning technology, arose in 1985 through the availability of specific TNF reagents [37]. Accordingly, as reviewed in [38], prior exposure to a passive antibody specific to TNF prevented illness in LPS-treated mice [39]. In due course excess TNF was also appreciated to be central to initiating inflammation as well as harmfully altering homeostasis of the normal physiology outlined in the previous paragraph. Information on all of these functional roles of TNF has continued to expand exponentially. As has been reviewed [40], the logic of the pathogenesis of certain infectious diseases being driven by TNF now encompasses essentially all such conditions [41–47]. Nowadays, as has often been reviewed, chronic sterile inflammatory states, such as rheumatoid arthritis (RA), psoriasis, Crohn's disease, late onset Alzheimer's disease (AD), and Parkinson's disease (PD), as well as the syndromes that follow survival from stroke and TBI, are also widely discussed in these terms.

## 4. Specific Anti-TNF Agents as Therapy against Noncerebral Disease

### 4.1. Inflammatory Disease That Resolves If Not Acutely Fatal

In the late 1980s, when enough rTNF had become available to see if it killed tumors in a clinical setting, the first firm indication of it being central to acute illness arose. But as predicted in the wider literature (above), it generated side effects that mimicked the onset of severe sepsis [48]. Yet while prior anti-TNF antibody prevented the illness caused by injecting LPS to induce experimental sepsis, it was of no help when administered to mice that had already been made sick by LPS [39]. This gave fair warning that such antibody was likely to be impractical for treating acute inflammatory disease once it is underway, and so it proved when etanercept, a major commercial anti-TNF biological agent, was first tested in sepsis patients [49]. This simply reflects the rapid kinetics of TNF release and clearance, which, in acute resolving diseases, often occurs before serious illness is apparent [50]. The antipathogen function of TNF [35], together with the large dose employed by present-day standards, plausibly explains outcomes being worse than controls in some sepsis groups [49].

Nevertheless certain acute iatrogenic states that mimic sepsis can be treated preemptively. As reviewed [40], a number of them have been confirmed to arise from excess production of TNF by appreciating that they can be prevented by prior use of specific anti-TNF agents. These include the Jarisch-Herxheimer reaction [51, 52], adverse reactions to OKT3 therapy [53, 54], and acute graft-versus-host disease [55–57]. Such conditions are not common, and preemptive avoidance of acute illness does not match dramatic cures for making headlines, but this preventative concept is accepted as logical within the wider clinical and TNF research communities.

### 4.2. Nonresolving Inflammatory Disease Approved for Treatment by Anti-TNF Agents

The challenge of disease states in which inflammation does not resolve is complex [58], but chronic conditions, in which production of proinflammatory cytokines persists, provide the opportunity for specific TNF agents to be useful therapy. RA was the first nonresolving inflammatory disease to be treated in this way, and the outcome, which opened many eyes, was pivotal in changing opinion on the usefulness of these agents. Crucially, a group working on RA reasoned that TNF was more important mediator than IL-1, since* in vitro* anti-TNF antibody reduced IL-1 as well as TNF [59, 60]. This prediction of anti-TNF superiority in this context was later fulfilled [61]. The implications of this for treating RA [62] led to a successful open trial with infliximab, the first of the commercial specific anti-TNF agents, being published a few years later [63]. With exemplary collaboration from the pharmaceutical company providing the drug and funding, a randomized double-blind trial that scaled up the 1993 open trial, retaining its route, dose, and timing, was published the following year [64] and led to the first registration of an anti-TNF agent for a human disease. Despite requiring fine tuning, this approach to treating RA still dominates the field and continues to change the face of this disease, as predicted 20 years ago [65]. Subsequently the use of specific anti-TNF agents became the optimal treatment for two other nonresolving inflammatory states, psoriasis [66] and Crohn's disease [67]. Hence 15 years ago three medical specialties involving three anatomically disparate sites, joints, skin, and gut lining, had become involved in the same treatment approach through acknowledging a shared fundamental disease mechanism.

As we have reviewed [68], the dual activity of TNF as a component of innate immunity and disease pathogenesis has made it inevitable that certain infections, particularly tuberculosis, have a tendency to be exacerbated during long-term anti-TNF therapy. The very extensive regular use of this treatment in a number of inflammatory diseases, particularly RA, demonstrates that this challenge can be managed successfully. It becomes much less of a concern when treatment is administered only once or twice, such as poststroke, as discussed below [69], compared to biweekly for RA.

## 5. Specific Anti-TNF Agents as Therapy against Central Nervous System Disease

As noted earlier, neuroscience knows the cytokine TNF through copious literature on its central roles in much normal brain physiology and, when in excess, chronic brain disease states in which function and behavior are altered [70, 71]. As reviewed [68, 72–76], within the past 10 years most aspects of the physiology and disease literature of the brain have felt its influence. This includes the spinal pain of sciatica, with a number of observational and controlled studies having employed etanercept to good therapeutic effect [77–81]. It might, therefore, have been reasonable to expect that treatment of conditions such as acquired brain injury by neutralizing excess TNF production would by now have followed due process and added another organ system to what is already a very successful strategy in the treatment of inflammatory disease at joints, skin, and gut. We need to examine why this has not happened. A conservative “silo” mentality, criticized within clinical neurology [3], is one possibility. Indeed, adoption of thrombolysis very soon after stroke onset, and now the present poststroke mainstay, was very vigorously opposed 20 years ago [82].

In large part, an explanation for this delay may lie in cerebral diseases being technically more difficult to treat because the specific anti-TNF agents currently in use, such as infliximab, etanercept, and adalimumab, are too large to pass through the blood-brain barrier in any meaningful amount after subcutaneous or intravenous administration except in massive experimental doses [83, 84]. Approaches under development to overcome this include TNF-specific nanobodies [85] small enough to pass the blood-brain barrier (BBB) (http://www.pharmatimes.com/article/12-06-26/Ablynx_plans_to_partner_RA_drug_lifted_by_immunogenicity_data.aspx), a “Trojan Horse” trans-BBB transport method [86, 87], and a third approach, the perispinal route, consisting of injecting etanercept into the cerebrospinal venous system, known also as Batson's plexus [88], followed by a head-down tilt for 5 minutes [89–91]. Perispinal administration is done on the argument that it allows, through reverse flow, rapid entry of large molecules into the CSF through the veins that usually drain this fluid [92, 93]. As proposed [69], the pattern, rapidity, and distribution of clinical responses best fit with rapid delivery of etanercept via retrograde distribution into the choroid plexus via the cerebrospinal venous system. Developing this last approach simply involved understanding the implications of the relevant anatomy [92–94] and considering it in the light of earlier aviation medicine research [95] on head-down tilting causing albumin and globulin, two etanercept-sized molecules in the plasma, to enter the CSF in appreciable amounts within five minutes.

The nanobody and Trojan Horse approaches have not yet generated human data, but the perispinal approach has done so, albeit only in open trial [89] and observational study [69, 96–98], spanning from 2006 to the present. Remarkably, no trial funding for the perispinal approach has yet been forthcoming, so random controlled trials for etanercept have been limited to subcutaneous injection [99, 100], clearly inadequate for a molecule this size to reach the CSF, therefore not surprisingly yielding negative results. This is despite far more documentation than was deemed sufficient, over 20 years ago, to trigger the first human therapeutic use of these agents—RA treatment with infliximab—to progress from open trial [63] to random double-blind trial [64] status within a year. The poststroke reports [69, 97, 98], consistent with extensive animal data [83, 84], have recently received favorable independent review [101]. In the absence, to date, of pharmaceutical company enthusiasm for random double-blind trials, individual patient pretreatment states have continued to act as recent historical controls [69]. This is in fact an ideal comparison, since the likelihood of rapid spontaneous return of function is remote so long after stroke events [102]. Wide variation in onset-to-treatment times, as well as phenotypical heterogeneity, is also overcome by using pretreatment controls. Moreover, it has recently been proposed that the degree of heterogeneity encountered in certain neurological diseases questions whether random blind trials are the best guide for individualized treatment decisions [103]. These authors argue in favor of observational studies alongside, complementing randomly controlled trials when groups are heterogeneous.

## 6. Considering Diseases by Common Pathophysiology rather than Anatomical Focus

The principle of encouraging rational therapy by grouping diseases under their pathophysiology rather than immediate cause or anatomical location was thoughtfully expanded in 2010, again from the perspective of the wide relevance of specific anti-TNF agents as rational therapy [104]. These authors pointed out that what they term as immune-driven inflammatory diseases (IMIDs) cover a wide range of medical specialities. They noted that classifying diseases by their pathophysiology would therefore better reflect emerging knowledge in disease mechanisms and more readily allow translation of new therapeutic concepts between anatomy-based specialist groupings. They also noted the advantage of using therapies that have been tested at length for safety and potential side effects on many people, in this case specific anti-TNF agents treating RA [64], making these agents of practical use in otherwise refractory diseases with high human and financial costs. The examples of specific anti-TNF agents being used in refractory sarcoidosis, refractory Behcet's disease, and refractory uveitis are referenced and discussed at length. These authors point to the usefulness of this reasoning in terms of responsibility to patients with conditions in which, for reasons such as phenotypic diversity, scarcity, or short life expectancy, recruitment into random controlled trials is impractical and off-label treatment warranted.

## 7. The Narrow Vision of the Traditional Anatomical Focus of Medical Specialties

Unfortunately, the current division of medicine into anatomically focused clinical specialties does not always help the above logic gaining a firm foothold. Arguably, this arises because some disease specialists schooled and experienced within a particular narrow, albeit deep, knowledge base rarely rub shoulders with those with expertise in cytokine biology, a key to understanding much disease pathogenesis across the board. The cytokine-disease link has now become a formidable body of information compared to its earlier size, when, for example, rheumatologist needed to assimilate this literature, so the challenge is now correspondingly larger, though not impossibly so, for new entrants such as neurologists. In contrast, cytokine scientists working on disease mechanisms need to cross anatomical boundaries in order to remain competitive and for decades have necessarily ignored conventional specialty barriers in their reading. Hence they are, in general, better equipped to accept the rationale of specific anti-TNF therapy for neuroinflammatory disease more readily and, having observed its effects here, advocate its further development [4–6].

Perhaps this relative lack of exposure to the broad cytokine literature is a large part of why this treatment concept has to date been dismissed by the clinical neurology community, despite not having observed its short- or long-term effects, as essentially impossible or dangerous [105, 106]. This may have contributed to a roadblock that noncerebral diseases, for which anti-TNF is established treatment, did not encounter a medical specialty, on which pharmaceutical companies depend for expert opinion on whether a certain random controlled trial is warranted, having, through refusal to observe, no informed opinion to offer. Importantly, this refusal, all the while protesting that the story cannot be taken seriously until random trials have been done, at present may, in part, be instrumental in deterring pharmaceutical company from funding these very trials. In such a climate, off-label treatment inevitably flourishes [104].

The point has been well made [3] that since inspiration can come from unexpected quarters, responsibility for the unmet needs of stroke patients can be said to require willingness to read the wider literature, as well as dispassionate first-hand consideration of any promising new approach from outside the expected medical specialty. The urgent need for this advice has recently been illustrated by a referenced, but unrefereed, website (http://www.sciencebasedmedicine.org/enbrel-for-stroke-and-alzheimers/) written by a clinical neurologist, recently being used to further the argument, in reference 33 of an American Academy of Neurology publication [100], that the concept of neutralizing excess TNF in a neurodegenerative condition is controversial. A key point argued on this site is that this single therapy could not plausibly treat several brain disease states (http://www.sciencebasedmedicine.org/enbrel-for-stroke-and-alzheimers/), since each has a different mechanism and so requires a different treatment. Current broad knowledge base of cytokine biology in disease would have allowed the neurologist author to appreciate that anti-TNF agents, already accepted as a logical treatment for RA, psoriasis, Crohn's disease, Jarisch-Herxheimer reaction, adverse reactions to OKT3 therapy, and acute graft-versus-host disease (see references in [40]), ankylosing spondyloarthritis and undifferentiated spondyloarthritis [107], sarcoidosis, uveitis, and Behcet's disease (see references in [104]), are also regarded, in the literature, as a logical approach to treating certain brain diseases, including disabilities consequent to traumatic brain injury, stroke, and other cerebral ischemic events. Other brain conditions in development for treatment with the same specific anti-TNF agents include the cognitive defects seen after surgery [108], after irradiation [109], after chemotherapy [110], and in sarcoidosis [111] and RA [112]. This breadth of literature dramatically demonstrates the wide spectra of disease in which the proinflammatory cytokines, such as TNF, which can also act as a homeostatic neurotransmitter in its own right [113], are implicated. The following list provides an overview of examples of specific anti-TNF biologicals in various stages of development or in use for treating disease:

 Systemic disease:
   rheumatoid arthritis [63, 64],
   psoriasis [66, 114, 115],
   Crohn's disease [116],
   spondyloarthritis [117],
   Jarisch-Herxheimer's disease [35, 51],
   graft-versus-host disease [56, 118],
   reaction to OKT-3 [53, 54].
 Chronic CNS disease:
   pain [77–79, 81, 113, 119–123],
   Alzheimer's disease [89, 124],
   Parkinson's disease [125],
   Huntington disease [126],
   post-LPS cognition [127],
   postoperative cognition [108],
   postirradiation cognition [109],
   postchemotherapy cognition [110],
   rheumatoid arthritis cognition [112],
   sarcoidosis cognition [111],
   poststroke therapy [69, 87, 97, 98],
   traumatic brain injury [69, 83].

Moreover, a serious student of cytokine biology would never regard specific anti-TNF agents simply as immunosuppressives (see above website), as if they were ibuprofen or aspirin. TNF in excess can be a proinflammatory cytokine but, as summarized earlier, it has many other activities that are as much a part of normal physiology as those of insulin and like it can become pathophysiological through loss of homeostasis [128]. In the same vein, excess TNF has many harmful actions unrelated to inflammation, such as the rapid inhibition of pain responses in the CNS by infliximab [113]. This study clearly differentiates between anti-TNF neutralizing the rapid direct effects of excess TNF and more slowly neutralizing inflammation once it has been set in train. Not having yet acquired this basic knowledge of cytokine biology presumably has influenced the main pharmaceutical company that markets etanercept to persist in its posted belief that the claimed rapidity of response [96] is biologically improbable because it is too fast for an inflammatory response to resolve (http://www.amgen.com/media/rapid_cognitive_improvement.html). This posting, still current at the time of writing, also ignores a number of confirmatory reports of a rapid response [4–6, 69, 97, 98, 129] since the initial text that noted it [96].

Other criticisms on the quoted (reference 33 of [100]) website critical of the perispinal approach, such as doubting a rationale for the reported improvement a range of years after the initiating event, can be addressed by engaging with neuroscientists, who can point to the extreme chronicity of cytokine excess that makes this plausible in stroke [130] and TBI [131, 132] survivors. Certainly, clinical neurology would have moved more in this direction if five years ago it had taken heed of key recommendation of the 53-authored* Stroke* editorial [3] that urges the field to be* alert to new models of disease that may vertically integrate basic, clinical, and epidemiological disciplines. For example, could advances in the understanding of infectious diseases or inflammation dramatically change our thinking about stroke pathogenesis?*

Clearly, understanding advances in infectious diseases and inflammation would take neurologists directly into the world of basic TNF biology and thence how the corrupting effects of its excessive poststroke production in the brain would lead to neurodegenerative disease. This points them to a treatment based on a logical, testable hypothesis, one amply demonstrated for some years in open trials and ripe for formal testing with etanercept. As summarized in this review, its prospects are far better than any other treatment, extant or proposed.

## Abbreviations

AD: Alzheimer's disease
BBB: Blood-brain barrier
BCG: Bacillus Calmette-Guérin
IMID: Immune-driven inflammatory disease
IP3R: Type-1 inositol trisphosphate receptors
LPS: Lipopolysaccharide
PD: Parkinson's disease
RA: Rheumatoid arthritis
TBI: Traumatic brain injury.
